# Intraoperative fluorescence imaging with aminolevulinic acid detects grossly occult breast cancer: a phase II randomized controlled trial

**DOI:** 10.1186/s13058-021-01442-7

**Published:** 2021-07-12

**Authors:** Kathryn Ottolino-Perry, Anam Shahid, Stephanie DeLuca, Viktor Son, Mayleen Sukhram, Fannong Meng, Zhihui ( Amy) Liu, Sara Rapic, Nayana Thalanki Anantha, Shirley C. Wang, Emilie Chamma, Christopher Gibson, Philip J. Medeiros, Safa Majeed, Ashley Chu, Olivia Wignall, Alessandra Pizzolato, Cheryl F. Rosen, Liis Lindvere Teene, Danielle Starr-Dunham, Iris Kulbatski, Tony Panzarella, Susan J. Done, Alexandra M. Easson, Wey L. Leong, Ralph S. DaCosta

**Affiliations:** 1 Princess Margaret Cancer Centre, University Health Network, Ontario Cancer Institute, 101 College Street, Toronto, M5G 1L7 Ontario Canada; 2 Laboratory Medicine Program, University Health Network, 200 Elizabeth Street, 11th Floor Eaton Wing, Toronto, M5G 2C4 Ontario Canada; 3 Biostatistics Department, University Health Network, 610 University Ave, Toronto, M5T 2M9 Ontario Canada; 4 Dermatology Department, Toronto Western Hospital, University Health Network, 399 Bathurst St, Toronto, M5T 2S8 Ontario Canada; 5 Department of Laboratory Medicine and Pathobiology, Faculty of Medicine, University of Toronto, 1 King’s College Circle, Toronto, M5S 1A8 Ontario Canada; 6 Surgical Oncology Department, Princess Margaret Cancer Centre of University Health Network, 610 University Ave, Toronto, M5T 2M9 Ontario Canada; 7 Department of Medical Biophysics, Faculty of Medicine, University of Toronto, 101 College Street, Toronto, M5G 1L7 Ontario Canada; 8 Techna Institute, University Health Network, 124-100 College Street, Toronto, Ontario M5G 1P5 Canada

**Keywords:** Breast cancer, Breast-conserving surgery, Fluorescence imaging, Intraoperative imaging, Aminolevulinic acid, Margin assessment, Optical imaging, Handheld intraoperative imaging device

## Abstract

**Background:**

Re-excision due to positive margins following breast-conserving surgery (BCS) negatively affects patient outcomes and healthcare costs. The inability to visualize margin involvement is a significant challenge in BCS. 5-Aminolevulinic acid hydrochloride (5-ALA HCl), a non-fluorescent oral prodrug, causes intracellular accumulation of fluorescent porphyrins in cancer cells. This single-center Phase II randomized controlled trial evaluated the safety, feasibility, and diagnostic accuracy of a prototype handheld fluorescence imaging device plus 5-ALA for intraoperative visualization of invasive breast carcinomas during BCS.

**Methods:**

Fifty-four patients were enrolled and randomized to receive no 5-ALA or oral 5-ALA HCl (15 or 30 mg/kg). Forty-five patients (n = 15/group) were included in the analysis. Fluorescence imaging of the excised surgical specimen was performed, and biopsies were collected from within and outside the clinically demarcated tumor border of the gross specimen for blinded histopathology.

**Results:**

In the absence of 5-ALA, tissue autofluorescence imaging lacked tumor-specific fluorescent contrast. Both 5-ALA doses caused bright red tumor fluorescence, with improved visualization of tumor contrasted against normal tissue autofluorescence. In the 15 mg/kg 5-ALA group, the positive predictive value (PPV) for detecting breast cancer inside and outside the grossly demarcated tumor border was 100.0% and 55.6%, respectively. In the 30 mg/kg 5-ALA group, the PPV was 100.0% and 50.0% inside and outside the demarcated tumor border, respectively. No adverse events were observed, and clinical feasibility of this imaging device-5-ALA combination approach was confirmed.

**Conclusions:**

This is the first known clinical report of visualization of 5-ALA-induced fluorescence in invasive breast carcinoma using a real-time handheld intraoperative fluorescence imaging device.

**Trial registration:**

Clinicaltrials.gov identifier NCT01837225. Registered 23 April 2013.

**Supplementary Information:**

The online version contains supplementary material available at 10.1186/s13058-021-01442-7.

## Introduction

Breast cancer, the most prevalent cancer in women, is often treated by breast-conserving surgery (BCS) [1, 2] which aims to completely excise the tumor with clear margins while preserving the maximum amount of healthy tissue. Cancer visualization under standard white light (WL) operating room conditions is difficult due to low cancer-to-normal tissue contrast, resulting in positive margins which are related to greater risk of local recurrence (LR) [3–6], inferior outcomes (complications, stress, poor cosmesis) [7], adjuvant delay [8], increased healthcare costs [9–11], and lower disease-specific survival [12]. Furthermore, incomplete resection requires reoperation in 20–70% of patients [2, 6, 13–20]. Thus, reducing positive margin rates is a major goal in BCS [21] and is an internationally recognized quality indicator for treatment [7, 22].

Currently, BCS best practice uses WL visualization, palpation, specimen radiography, and intraoperative histopathology to guide resection. These techniques are lengthy (~ 20 min) [23], limited by inaccurate co-localization of positive margins on the excised tissue to the surgical bed [24] and variably impact outcomes [23]. Adaptation of standard medical imaging technologies for operating room use (MRI [25], ultrasound [25, 26], PET/CT [25]), and emerging intraoperative tumor detection technologies for BCS, both non-optical and optically enabled, are either at the preclinical stage or are not widely adopted due to practical limitations. Thus, there is a clinical need for an alternative, safe, practical, cost-sensitive, and real-time intraoperative imaging technology for surgeons and pathologists to visualize occult malignancy in excised specimens and surgical cavities during index BCS.

Fluorescence imaging using an intraoperative instrument plus an exogenous tumor-specific imaging agent may fulfill this need by enhancing tumor tissue contrast and facilitating the detection of grossly occult disease. Intraoperative fluorescence imaging for detection of carcinoma has been demonstrated in clinical trials for several tumor types using visible and near-infrared (NIR) contrast agents [27–30]. However, the instrumentation involves large, costly cart-based systems that are impractical for BCS since they do not fulfill the surgeon’s need to interrogate both the surgical specimen and cavity. To address this, we developed a handheld fluorescence imaging device (Portable Real-time Optical Detection Identification and Guide for Intervention, PRODIGI) for real-time intraoperative fluorescence imaging of excised breast specimens and the surgical cavity. PRODIGI is clinically safe and has demonstrated clinical utility in other medical applications [31–33]. The device combines consumer-grade imaging sensor technology with miniature light-emitting diodes (LEDs) and multiband optical filtering to capture high-resolution WL and fluorescence digital images and videos. PRODIGI’s multiband optical filter allows for imaging of porphyrins, including protoporphyrin IX (PpIX), which fluoresces red (peak emission at 635 nm wavelength) [34, 35] when excited by violet-blue light (~ 400–410 nm). PpIX is a metabolite of the prodrug 5-aminolevulinic acid (5-ALA), an endogenous non-protein amino acid involved in heme biosynthesis in mammalian cells. Unlike contrast agents that are receptor-targeted or enzyme-activated, PpIX is not affected by suboptimal biodistribution and tumor penetration, heterogenous target receptor/enzyme expression [29], and high background fluorescence [29, 36].

Delivered systemically, 5-ALA is taken up by cells throughout the body where it is converted into heme. In aberrant cells, such as cancer cells, defects in heme biosynthesis cause accumulation of PpIX [35, 37–42], enabling real-time visualization. 5-ALA is widely known for its clinical use in photodynamic diagnosis [43–55] and therapy [34, 49, 56, 57] of premalignant and malignant disease. Large clinical trials have demonstrated the safety and clinical utility of 5-ALA-based fluorescence image-guided surgery (FIGS) for malignant glioma [58–61], which led to the approval of oral 5-ALA hydrochloride (HCl) for FIGS of high-grade glioma in approximately 40 countries, including the USA [62]. Other clinical studies support 5-ALA for the visualization of malignant tissue in the bladder [44], gastrointestinal tract [50], oral cavity [51, 52], lung [47, 55], and female genital tract [49, 53, 54]. 5-ALA has a well-established safety profile, which is highlighted in the Gleolan Prescribing Information for glioma patients where pyrexia, hypotension, nausea, and vomiting are reported to have occurred in > 1% of patient’s in the week following surgery and chills, photosensitivity reaction, solar dermatitis, hypotension, abnormal liver function test, and diarrhea are reported to have occurred in < 1% of patients in the 6 weeks after surgery [62]. Only one other clinical study (in 2001) explored the utility of 5-ALA (40 mg/kg bodyweight (BW)) for detecting breast cancers in a non-randomized study of 16 patients, although the results were exploratory and limited to imaging surgical specimens only [48]. Since then, no follow-up studies on the application of 5-ALA in breast cancer imaging have been published.

In this report, we present the results of a single-center non-interventional Phase II randomized controlled trial (RCT) designed to characterize the imaging performance of our handheld imaging device with two doses (15 and 30 mg/kg BW) of 5-ALA HCl versus no tumor contrast in patients with invasive breast cancer undergoing lumpectomy or mastectomy. Two doses (15 or 30 mg/kg BW) of 5-ALA HCl were administered to evaluate the minimum dosage for sufficient tumor-to-normal tissue fluorescence contrast obtained using the PRODIGI device. The doses selected are “on either side” of the FDA-approved dose for glioma (20 mg/kg) [62] and are based on previous clinical studies validating safety at ≤ 60 mg/kg [63–65], tumor enhancement as low as 10 mg/kg [59, 60, 64], and improved tumor-to-normal tissue contrast with increased doses [60, 64]. The primary objective was to determine measures of diagnostic accuracy (positive and negative predictive values, sensitivity, specificity, and diagnostic odds ratio) of visualization of breast tumors in surgical specimens using PRODIGI in combination with 5-ALA HCl, with ex vivo biopsy-based histology as the gold standard. The secondary objective was to evaluate the feasibility and safety of the PRODIGI device for intraoperative imaging of the surgical cavity following resection. Finally, we confirm the safety and technical feasibility and discuss the future clinical adoptability of PRODIGI plus 5-ALA HCl to improve tumor visualization during BCS.

## Materials and methods

### Study design

The Princess Margaret Cancer Center (PMCC; Toronto, Canada), University Health Network (UHN) Research Ethics Board (REB#10-0633-CE) and Mount Sinai Hospital (MSH; Toronto, Canada) Research Ethics Board (REB#13-0155E) approved the clinical protocol for this study (clinicaltrials.gov identifier NCT01837225). All subjects provided written informed consent and were randomized to receive no 5-ALA, 15, or 30 mg/kg 5-ALA ~ 3 h before surgery (2–4 h prior to anesthesia). A randomization table was generated before study initiation using the List Randomization tool from RANDOM.org and stored in a locked cabinet. The primary objective of this RCT was to determine measures of diagnostic accuracy of PpIX fluorescence for visualizing breast tumors in patients receiving oral 5-ALA HCl. Participants underwent standard breast cancer surgery (lumpectomy or mastectomy) by one of two study surgeons (A.M.E., W.L.L.), followed by fluorescence imaging of the cavity and surgical specimen by a researcher, and collection of biopsies from the surgical specimen by the pathologist’s assistant (PA) (V.S., M.S., F.M.). The first 5 patients randomized per group underwent ex vivo imaging of the surgical specimen only. Imaging of the surgical cavity and specimen was performed in the remaining 10 patients/group. Neither the surgeon (A.M.E., W.L.L.), researcher, nor PA (V.S., M.S., F.M.) were blinded to allocation.

Imaging was performed en face of the external surface of the intact surgical specimens followed by the serially sliced specimen (1–1.5 cm thickness/slice). Biopsies were collected from two spatially distinct areas of specimen slices: (i) inside the grossly demarcated primary tumor border near the center of the tumor and (ii) outside the grossly demarcated primary tumor border (see Study Workflow, Supplementary Fig. 1). Biopsies were collected from areas of red and non-red fluorescence tissue to determine the accuracy of fluorescence visualization of clinically obvious (inside the tumor border) and clinically occult (outside the tumor border) disease. A pathologist (S.J.D.) blinded to fluorescence imaging results evaluated biopsies for the presence of cancer (including invasive and in situ disease). Surgeons (A.M.E., W.L.L.) and pathologists (S.J.D.) did not modify their conventional margin assessment based on fluorescence imaging results or use fluorescence imaging to guide resection.

### Study population

**Fig. 1:**
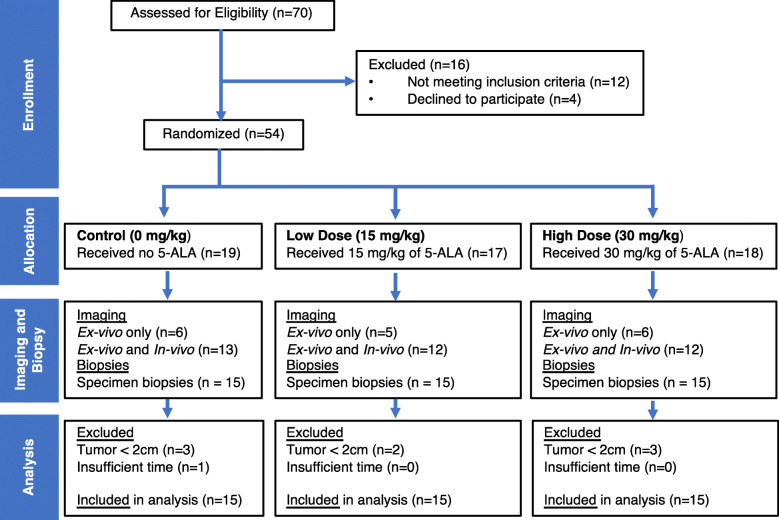
CONSORT diagram. Patients with tumors measuring < 2 cm (greatest dimension) at specimen gross examination were excluded from the analysis because biopsies could not be collected from inside the demarcated tumor border. One case was excluded because of insufficient time to complete data collection prior to initiating formalin fixation of the specimen

Patients were screened for eligibility by their breast cancer surgeon (A.M.E., W.L.L.) during their pre-operative clinic visits. The inclusion criteria were as follows: ≥ 18 years of age, female, diagnosed with invasive breast cancer, primary tumor measuring ≥ 2 cm in diameter based on diagnostic imaging, consented to standard surgeries for primary invasive breast cancer with/without axillary procedure (axillary dissection or sentinel node biopsy), and consented to banking of core biopsies with UHN tissue bank. The exclusion criteria were as follows: pre-operative therapy (chemotherapy, endocrine therapy, radiotherapy), history of photosensitivity, liver disease or recurrent disease, pregnant, tumor diameter < 2 cm at grossing (as stipulated by the pathology department), unable to consent, or refused tissue banking. Seventy patients were consented to participate in the study, of which 14 were withdrawn prior to randomization and 9 after randomization (Fig. 1) due to the following reasons: tumor diameters measuring < 2 cm at the time of gross examination, change of treatment plan to include pre-operative chemotherapy or surgery at a hospital other than PMCC or patient choice. Data from withdrawn participants was not included in the patient demographics or data analysis.

### 5-ALA HCl doses and administration

5-ALA HCl (generously provided by photonamic GmbH and Co. KG, Pinneberg, Germany) was dissolved in tap water and administered orally by a study nurse 2–4 h before surgery in patients randomized to the 15 or 30 mg/kg BW 5-ALA groups. Precautionary measures were taken to prevent adverse events related to 5-ALA, including monitoring for symptoms related to skin photosensitivity [63–65] and providing homecare instructions to minimize exposure to sunlight and/or bright indoor light for at least 48 h following 5-ALA ingestion. Patients were provided with SPF 60 sunscreen and instructed how to use it prior to leaving the hospital. Patient records were reviewed for any adverse events reported at the post-surgical follow-up with their surgeon (A.M.E., W.L.L.).

### Handheld fluorescence imaging device

PRODIGI (Supplementary Fig. 2) is a handheld fluorescence imaging device prototype developed by our group for clinical fluorescence imaging applications [60, 61]. PRODIGI emits 405 nm light with an optical power density of 1.14 mW/cm^2^at a 10-cm imaging distance. Fluorescence imaging was performed with all room lights turned off and intrinsic camera sensor settings (e.g., ISO: Auto; Exposure Value: 0; Macro: Auto) were kept the same across all image acquisition sessions. The device was cleaned with disinfecting wipes between uses. Images were transferred to a designated password-protected personal computer after each imaging session for storage and analysis. The device was Canadian Standards Association certified for electrical safety prior to use and exempt from Health Canada Investigational Testing Authorization requirements.

### In vivo fluorescence imaging of the surgical cavity

The technical feasibility, safety, and integration into the clinical workflow of in vivo imaging of the surgical cavity immediately following lumpectomy or mastectomy was examined using PRODIGI in n = 30 (n = 10/group) patients. The device and its power supply cable were covered in a disposable sterile plastic drape (Laser Arm Drape, Cardinal Health, 29-59029) using an instrument draping protocol to maintain sterility. Small neodymium magnets permanently embedded in the device were attached to four autoclaved magnets on the exterior of the drape thereby holding the drape flush against the emission filter. The draping protocol complied with the safety requirements of the International Electrotechnical Commission IEC 60601-1 such that heat generated from the LEDs was safely dissipated within the drape during use. Real-time still and video WL and fluorescence imaging of the cavity was performed by a member of the research team with the assistance of the surgeon (A.M.E., W.L.L.), who manipulated the cavity to expose the entire inner surface of the cavity. Overhead room lights and surgical lights were turned off during fluorescence imaging.

### Ex vivo specimen preparation, image acquisition, and interpretation

Following breast cancer surgery, surgical specimens were sent to pathology where WL and fluorescence images were acquired of all anatomical surfaces of the intact specimen. Margins were painted by the PA (V.S., M.S., F.M.) using a standardized margin inking schema (different colors for different margins) following fluorescence imaging of the intact specimen, to avoid ink artefacts. Specimens were serially sliced grossly (“bread-loafed”) [66] and the slice containing the largest clinically discernable cross-sectional of tumor was laid open. A digital WL image of the complete slice was transferred to a tablet computer (Galaxy Note, Model GT N8010, Samsung). WL images were indelibly marked by the PA (V.S., M.S., F.M.) using Photoshop (Adobe) to demarcate the clinically identifiable primary tumor border based on visualization and palpation. In cases of nonpalpable cancers or tumors with an ill-defined edge, no primary tumor border was marked. Fluorescence images were collected to ensure each WL image had a spatially colocalized fluorescence image. Specimen imaging was performed with the device held ~ 10 cm from the tissue. For scale, a white specimen sticker was used during imaging. Images were date stamped and saved for analysis. Fluorescence images were acquired under low light, consistent across imaging sessions. In some patients, additional WL and fluorescence images of adjacent specimen slices and slices identified by the PA (V.S., M.S., F.M.) as “tumor-free” were collected. WL and fluorescence image acquisition took < 1 s and 1–2 s, respectively per surface or specimen slice. Specimens were placed in formalin for fixation within 1 h of surgical excision as per clinical practice.

### Tissue biopsy collection and analysis

Research tissue biopsies were collected by study research staff from ex vivo specimens of all 45 patients (n = 141 biopsies) for gold standard histological evaluation by the study pathologist (S.J.D.) blinded to the imaging results. A 2- or 4-mm diameter punch biopsy device (Cat #12-460-409 and -399, Thermo Fisher Scientific, New Hampshire, USA) was used. Biopsies were collected from inside and outside the tumor border. Outside the border, fluorescence imaging was used to target biopsy collection in areas with red or no-red PpIX fluorescence. A minimum of 1 biopsy (inside the tumor border, to retain sufficient carcinoma for clinical diagnosis) and a maximum of 4 biopsies (tumor plus additional biopsies, to ensure specimen fixation commenced within an acceptable ischemia time) were collected per specimen (total biopsies = 141). Most biopsies (n = 134) were fixed in neutral buffered formalin, paraffin-embedded, sectioned (4 μm), and H&E stained. A subset of tumor biopsies (n = 7) were placed in a tissue cassette, immersed in optimal cutting temperature compound, flash frozen in liquid nitrogen, and protected from light using established methods before fluorescence microscopy visualization of the cellular localization of PpIX fluorescence. All H&E sections were evaluated by the blinded study pathologist (S.J.D.) for invasive and/or in situ cancer. When required, immunohistochemical stains (High Molecular Weight Keratin, Estrogen Receptor, Calponin, Smooth Muscle Myosin, CAM5.2, P63) were performed on serial sections according to standard institutional staining protocols. We used a custom quantitative fluorescence (qF) system [38] to characterize the fluorescence spectra between 500 and 800 nm emission (405 nm excitation) and measure local tissue PpIX concentration at locations within the primary tumor border before biopsy. Tissue fluorescence spectra were obtained by a fiberoptic probe connected to a bench-top spectrometer, and a validated algorithm was used to calculate absolute PpIX concentrations [38].

### Ex vivo tissue fluorescence microscopy

A subset of biopsies (n = 7) from inside the primary tumor border were processed by frozen section for fluorescence microscopy. Frozen biopsies were collected from n = 4 low-dose patient specimens and n = 3 high-dose patient specimens. Frozen biopsies were cut serially into 8 μm frozen tissue sections and placed on glass microscopy slides for confocal fluorescence microscopy before histological staining. Frozen sections were wrapped in tin foil to limit light exposure and stored at − 80 °C until fluorescence microscopy was performed. Serial sections were cut from each biopsy for confocal microscopy, H&E, and additional staining, as required. Slides were imaged with the Nikon A1R resonance scanning confocal fluorescence microscope (Nikon Instruments Inc. Melville, NY, U.S.A.) using 405 nm excitation, a 525/50-nm emission filter for background tissue autofluorescence (AF) and a 600/50 + 685/70-nm emission filter for PpIX fluorescence. Resonance mode was used to scan frozen tissue sections rapidly (30 or 420 frames/second), to optimize image acquisition settings and minimize photobleaching of PpIX fluorescence. Fluorescence images were acquired using Galvano scanning at 1024 × 1024 pixels.

Immediately following fluorescence microscopy, frozen sections were fixed in formalin and stained with H&E. Two additional serial sections of the same sample were stained using standard protocols with Masson’s trichrome (MT) to identify connective tissues and Oil Red O (ORO) to identify adipocytes. This enabled spatial correlation between fluorescent tissue features in the frozen section and tissue-specific staining for histology. Nikon NIS Elements C software (Nikon Instruments Inc. Melville, NY, USA) was used to convert fluorescence microscopy images to TIF format and Aperio ImageScope was used to export corresponding images of H&E, MT, and ORO-stained tissues to JPEG image format. Fluorescence H&E, MT, and ORO-stained tissue section images were examined by the study pathologist (S.J.D.).

### Image analysis of stained tissue sections

Stained biopsy tissue sections from 5-ALA patient specimens were digitalized using the Aperio Scanscope XT whole-slide scanner (Aperio Technologies, Inc., Vista, CA, USA). HALO Image Analysis software v2.0.1145.14 (Indica Labs, Albuquerque, NM, USA) was used to quantify the area and relative amount tissue types (cancer, connective, adipose) within biopsies histologically confirmed to contain cancer cells based on pathologist (S.J.D.)-blinded examination. A blinded researcher used the HALO Classifier Module to train the proprietary machine-learning algorithm to identify and differentiate tissues based on differences in color, texture, and contextual features. Previous work has validated this software for detecting pathology in human paraffin-embedded tissue [67]. Up to four different classifiers were defined per tissue section: carcinoma, connective, adipose tissue, and non-tissue area (background). Areas corresponding to each tissue type were manually highlighted to provide training inputs and refined for each section with additional training inputs to improve accuracy. Cancer cells, stromal tissue, and adipocytes were identified with the aid of the Classifier Module and the area (mm^2^) for each tissue type as well as the total biopsy slice area was obtained based on pixel algorithms. The percentage of cancer tissue (area of cancer pixels/area of tissue pixels) and connective tissue (area connective tissue pixels/area of tissue pixels) was calculated, as well as the ratio of tumor over connective tissue (area of tumor pixels/area of connective tissue pixels). Aperio ImageScope (Aperio Technologies, Inc., Vista, CA, USA) was used to export images of H&E-stained sections in JPEG format for publication.

### Analyses of clinical fluorescence images

Fluorescence images of serially sliced specimens were analyzed using Matlab v2018b (The MathWorks, Inc., Massachusetts, USA). Slices annotated with the primary tumor border were included (n = 14, control; n = 11, low dose; n = 12, high dose). During gross examination, the PA (V.S., M.S., F.M.) identified the primary tumor of 8 specimens as having ill-defined borders and the images were not annotated with the clinically demarcated primary tumor border. These 8 specimens were excluded from this analysis. JPEG images were converted from RGB to XYZ format using an inbuilt Matlab function. Regions of interest corresponding to the primary tumor and normal tissue were defined based on the clinically demarcated primary tumor border. Background areas without tissue or that included the scale bar sticker, and areas outside the primary tumor that had histologically confirmed cancer were excluded. The average X, Y, and Z values for all pixels in the primary tumor ROI and normal tissue ROI were calculated, converted into (x, y)^tumor^ and (x, y)^normal^ coordinates, and plotted on a CIE chromaticity graph. The Euclidean distance between (x, y)^tumor^ and (x, y)^normal^, which corresponds to a perceived difference in color according to the MacAdams diagram (Supplementary Fig. 3), was calculated per image using an inbuilt Matlab program. The average ± standard deviation Euclidean distance for images from patients in each group was compared.

### Statistical analysis

Specimen fluorescence images and corresponding research tissue biopsies (n = 93) from patients treated with 5-ALA were used to estimate the PPV, NPV, sensitivity, specificity, and diagnostic odds ratio (DOR) of the device plus 5-ALA to differentiate between breast cancer and healthy tissues. The exact method (Pearson-Clopper) was used to calculate the 95% confidence intervals of the estimated PPV, NPV, sensitivity and specificity, and normal approximation on logarithmic scale was used for the confidence intervals of the DOR. True positives were biopsies from a PpIX red fluorescence area, histologically confirmed as cancer cell positive (invasive and/or in situ), whereas false positives were biopsies from a red fluorescence area, histologically confirmed as cancer cell negative. True negatives were biopsies from a non-(PpIX) red fluorescence area, histologically confirmed as cancer cell negative, whereas false negatives were biopsies from a non-red fluorescence area, histologically confirmed as cancer cell positive. PPV is defined as the probability of cancer being present in an area of tissue identified as positive for PpIX red fluorescence, and NPV is defined as the probability of cancer being absent in an area of tissue identified as negative for PpIX red fluorescence. DOR is a measure of test performance defined as the ratio of the odds of detecting red PpIX fluorescence if cancer is present relative to the odds of detecting red PpIX fluorescence if cancer is not present. Scatterplots and Mann-Whitney tests were used to compare proportions of cancer and connective tissue, and the ratio of cancer and connective tissue pixels between biopsies from red fluorescence regions. Bar graphs and one-way ANOVA with Tukey’s multiple comparisons test were used to compare Euclidean distances between all groups. R v3.4.1 (R Core Team 2017. R: A language and environment for statistical computing. R Foundation for Statistical Computing, Vienna, Austria) was used to calculate diagnostic measures of accuracy and GraphPad Prism v8 (GraphPad Software, Inc. La Jolla, CA) was used to generate graphs and perform Mann-Whitney and ANOVA tests.

## Results

### Subject demographics

Forty-five females (29–83 years old) undergoing lumpectomy (n = 29) or mastectomy (n = 16) for primary ductal (n = 37), lobular (n = 7), or mixed (n = 1) invasive carcinoma at the Princess Margaret Cancer Center (PMCC, Toronto, Ontario, Canada) between August 2013–August 2018 were included in the analysis (Table 1). Patients were randomized 1:1:1 to receive no 5-ALA (control), 15 mg/kg (low dose) or 30 mg/kg (high dose) BW orally administered 5-ALA 2-4 h before surgery. Ten patients (22.2%) had margins revised at the surgeons’ (A.M.E., W.L.L.) discretion during index surgery. Seven (15.6%) patients had at least one involved margin on the index surgical pathology report, of which 6 (13.3%) were positive for invasive carcinoma and 2 (4.44%) were positive for ductal carcinoma in situ (DCIS). Six of 45 patients (13.3%) underwent a second operation to achieve negative margins.

**Table 1:** Baseline characteristics and surgical procedures

	Control (***n*** = 15)	Low Dose (***n*** = 15)	High Dose (***n*** = 15)	Total (***n*** = 45)
**Age (years) (mean, SD)**	55.5 (15.3)	57.5 (13.2)	53.8 (9.9)	55.6 (12.8)
**Surgery Type (N, %)**				
Lumpectomy	10 (66.7)	9 (60.0)	10 (66.7)	29 (64.4)
Mastectomy	5 (33.3)	6 (37.5)	5 (33.3)	16 (35.5)
**Diagnosis (N, %)**				
IDC	13 (86.7)	13 (86.7)	11 (73.3)	37 (82.2)
ILC	1 (6.7)	2 (13.3)	4 (26.7)	7 (15.6)
IMC	1 (6.7)	0 (0.0)	0 (0.0)	1 (6.7)
**Primary Tumour Size (cm) (mean, SD)**	3.1 (1.2)	2.7 (0.9)	3.3 (2.2)	3.0 (1.5)
**Grade (N, %)**				
1	1 (6.7)	1 (6.7)	1 (6.7)	3 (6.7)
2	4 (26.6)	6 (40.0)	5 (33.3)	15 (33.3)
3	10 (66.7)	8 (53.3)	9 (60.0)	27 (60.0)
**Mitotic Score (N, %)**				
1	4 (26.7)	5 (33.3)	5 (33.3)	14 (31.1)
2	3 (20.0)	3 (20.0)	4 (26.7)	10 (22.2)
3	8 (53.3)	7 (46.7)	6 (37.5)	21 (46.7)
**ER Status (N, %)**				
Positive	12 (80.0)	12 (80.0)	11 (73.3)	35 (77.8)
Negative	3 (20.0)	3 (20.0)	4 (26.7)	10 (22.2)
**PR Status (N, %)**				
Positive	12 (80.0)	10 (66.7)	10 (66.7)	32 (71.1)
Negative	3 (20.0)	5 (33.3)	5 (33.3)	13 (28.9)
**Her2 Status (N, %)**				
Positive	1 (6.7)	4 (26.7)	2 (13.3)	7 (15.6)
Negative	14 (93.3)	11 (73.3)	13 (86.7)	38 (84.4)
**DCIS Present (N, %)**				
Yes	12 (80.0)	10 (66.7)	9 (60.0)	31 (68.9)
No	3 (20.0)	5 (33.3)	6 (37.5)	14 (31.1)
**Margin Status (N, %)**				
Positive (Invasive)	1 (6.7)	1 (6.7)	4 (26.7)	6 (13.3)
Negative (Invasive)	14 (93.3)	14 (93.3)	11 (73.3)	39 (86.7)
Positive (DCIS)	1 (6.7)	0 (0.0)	1 (6.7)	2 (4.4)
Negative (DCIS)	14 (93.3)	15 (100)	14 (93.3)	43 (95.6)
**Revised Margins at Index Surgery (N, %)**^**a**^				
Yes	3 (20.0)	3 (20.0)	4 (26.7)	10 (22.2)
No	12 (80.0)	12 (80.0)	11 (73.3)	35 (77.8)
**Re-excision (N, %)**				
Yes	2 (13.3)	1 (6.7)	3 (20.0)	6 (13.3)
No	13 (86.7)	14 (93.3)	12 (80.0)	39 (86.7)

*SD* standard deviation, *IDC* invasive ductal carcinoma, *ILC* invasive lobular carcinoma, *IMC* invasive mammary carcinoma, *ER* estrogen receptor, *PR* progesterone receptor, *HER2* Human epidermal growth factor receptor 2, *DCIS* ductal carcinoma *in situ*

^a^margin revision not guided by fluorescence imaging

In patients receiving 5-ALA, oral administration occurred between 2 and 4 h before anesthesia with an average time to imaging ± SD of 248.3 ± 55.8 min *vs* 246.1 ± 37.3 min (p = 0.90, t-test) for the low- and high-dose 5-ALA groups, respectively.

### 5-ALA-induced fluorescence in clinically obvious and clinically occult disease

**Fig. 2:**
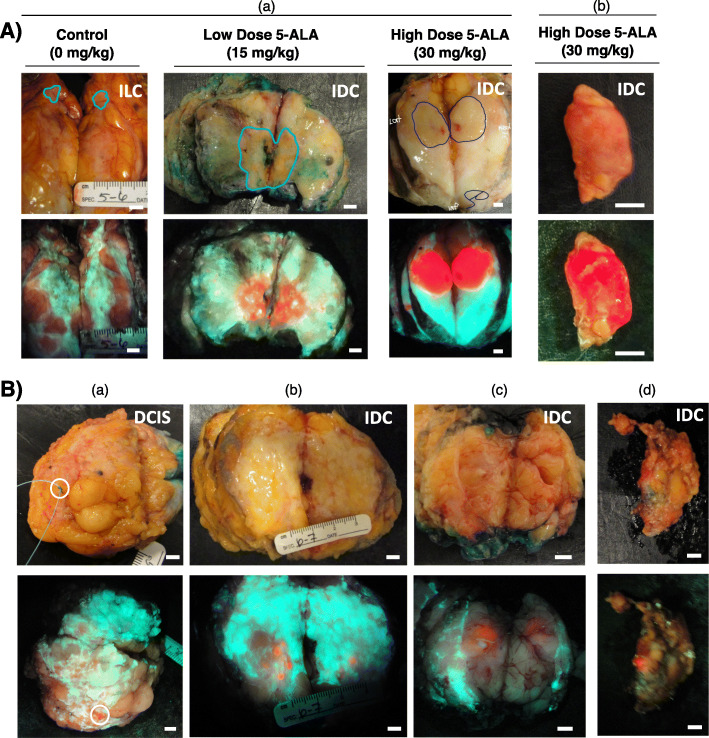
Fluorescence imaging of 5-ALA-induced PpIX fluorescence in grossly obvious and grossly occult carcinoma. **A** Representative white light (top row) and fluorescence (bottom row) images of grossly obvious disease in sectioned lumpectomy specimens (a) and a clinically positive sentinel lymph node (c). The pathologist’s assistant (V.S., M.S., F.M.) demarcated tumor border (blue line) identified the grossly obvious tumor in the sectioned specimens. The surgeon (A.M.E., W.L.L.) identified the lymph node as grossly obvious for disease. **B** Representative white light (top row) and fluorescence (bottom row) images of grossly occult disease at the surface of an excised lumpectomy (a), in grossly sectioned specimens (b, c) and a sentinel lymph node (d) from patients with invasive ductal carcinoma with (a, d) or without (b, c) a DCIS component administered 15 mg/kg (b, c) or 30 mg/kg (a, d) 5-ALA HCl. Images represent tissue that was identified by the surgeon (A.M.E., W.L.L.) (a, d) or pathologist’s assistant (V.S., M.S., F.M.) (b, c) as grossly negative for the presence of cancer. (a) DCIS identified by fluorescence imaging at the lumpectomy margin. (b, c) Invasive carcinoma identified by fluorescence imaging on slices outside the grossly demarcated tumor. (d) Invasive carcinoma macro-metastases identified by fluorescence imaging in an excised sentinel lymph node. Scale bars = 5 mm

Bright red PpIX fluorescence was detected inside the demarcated tumor border in grossly sectioned specimens only from patients administered either 5-ALA dose (Fig. 2)*.* PpIX fluorescence was observed in both invasive ductal carcinoma (IDC; with or without DCIS) and invasive lobular carcinoma (ILC). In the absence of 5-ALA, tumor AF was indistinguishable from background normal adipose and connective tissue AF.

Strong PpIX fluorescence was detected in grossly obvious disease (Fig. 2A), which was visibly appreciable and/or palpable both in grossly sectioned surgical specimens (Fig. 2A(a)) and clinically positive tumor-draining lymph nodes (Fig. 2A(b)). The boundary between the PpIX fluorescence primary tumor and surrounding normal tissue of the grossly sectioned specimen was well-defined and aligned closely with that of the pathologist’s assistant (PA) (V.S., M.S., F.M.) demarcated tumor border (Fig. 2A(a)). 5-ALA-induced PpIX fluorescence was also detected in grossly occult disease, which was not otherwise obvious under intraoperative standard of care (Fig. 2B). Grossly occult disease, including IDC and DCIS, was detected by fluorescence imaging on the margin of the intact specimen (Fig. 2B(a)), in sectioned surgical specimens (Fig. 2B(b-c)) and in tumor-draining lymph nodes (Fig. 2B(d)).

### Diagnostic accuracy of fluorescence imaging with 5-ALA HCl

Diagnostic measures of accuracy were calculated to evaluate the ability of 5-ALA-induced PpIX red fluorescence to discriminate breast carcinoma using the PRODIGI device. Blinded pathologist (S.J.D.) analysis of hematoxylin and eosin (H&E)-stained research biopsies served as the gold standard for comparison with fluorescence imaging results. Control tumors (no 5-ALA) appeared as green and pink/brown fluorescence and lacked perceptible fluorescence contrast to differentiate tumors from surrounding healthy tissue (Fig. 2A(a)). As a result, all biopsies collected from control patients (n = 47) were classified as negative for PpIX red fluorescence (except a single biopsy, Supplementary Fig. 4). Diagnostic measures of accuracy to detect PpIX red fluorescence tumors are therefore not presented for the control group. Data for the low-dose (n = 46 biopsies) and high-dose (n = 48 biopsies) 5-ALA groups are presented in Table 2. Measures were calculated separately for the areas inside and outside the grossly demarcated tumor border, accounting for the anticipated difference in cancer prevalence in these areas. The positive predictive value (PPV) was 100.0% for both the 15 and 30 mg/kg groups inside the tumor border. Outside the tumor border, the PPV was 55.6% and 50.0% for the low- and high-dose groups, respectively. The NPV inside the tumor border is not reported since the prevalence of normal healthy tissue inside the tumor border is expected to be extremely low and therefore the denominator of NPV is expected to be near zero regardless of the results of the fluorescence imaging. However, outside the demarcated tumor border, the NPV was 95.5% and 90.9% for the low- and high-dose groups, respectively.

**Table 2:** Performance of fluorescence imaging device to detect carcinoma in patients administered 5-ALA stratified by biopsy location. Measures of diagnostic accuracy were calculated separately for the areas inside and outside the demarcated tumor border. Areas of fluorescence images classified as negative or positive for PpIX red fluorescence (−Red FL/+Red FL) were biopsied and fluorescence imaging results were compared to gold-standard histological evaluation of biopsy H&E tissue sections performed by a blinded pathologist (S.J.D.)

	Low Dose (15 mg/kg)				High Dose (30 mg/kg)			
	Inside the tumor border		Outside the tumor border		Inside the tumor border		Outside the tumor border	
	-Cancer	+Cancer	-Cancer	+Cancer	-Cancer	+Cancer	-Cancer	+Cancer
-Red FL	1	6	21	1	0	5	20	2
+Red FL	0	8	4	5	0	10	5	5
PPV % (95%CI)	100.0% (63.1 – 100.0)		55.6% (21.2 – 86.3)		100.0% (73.5 – 100.0)		50.0% (18.7 – 81.3)	
NPV % (95%CI)	N/A		95.5% (77.2 – 99.9)		N/A		90.9% (70.8 – 98.9)	
Sensitivity % (95%CI)	57.1% (28.9 – 82.3)		83.3% (35.9 – 99.6)		66.7% (38.4 – 88.2)		71.4% (29.0 – 96.3)	
Specificity % (95%CI)	100.0%^a^ (2.5 – 100.0)		84.0% (63.9 – 95.5)		N/A^b^		80.0% (59.3 – 93.2)	
DOR (95%CI)	N/A		26.3 (2.38 – 288.94)		N/A		10 (1.5 – 67.6)	

*FL* fluorescence, *PPV* positive predictive value, *NPV* negative predictive value, *CI* confidence interval, *DOR* diagnostic odds ratio, *N/A* not applicable

^a^a single tumor negative biopsy was collected inside the demarcated tumor border

^b^no tumor negative biopsies collected inside the tumor border

The specificity outside the tumor border was 84.0% and 80.0% in the low- and high-dose groups, respectively. The DOR was 26.3 and 10 for the 15 and 30 mg/kg groups, respectively. The measures of diagnostic accuracy for all biopsies, irrespective of collection location, are presented in Table 3. Overall, sensitivity and specificity to detect cancer was 65.0% and 84.6%, respectively in the low dose group and 68.2% and 80.0%, respectively in the high-dose group. The PPV was 76.5% (low dose) and 75.0% (high dose) and the NPV was 75.9% (low dose) and 74.1% (high dose). No significant difference in the measures of diagnostic accuracy was observed between the low- and high-dose groups (Supplementary Table 1).

**Table 3:** Performance of fluorescence imaging device to detect carcinoma in patients administered 5-ALA. Overall measures of diagnostic accuracy were calculated irrespective of the biopsy location (analysis combined biopsies collected inside and outside the tumor border). Areas of fluorescence images classified as negative or positive for PpIX red fluorescence (−Red FL/+Red FL) were biopsied and fluorescence imaging results were compared to gold-standard histological evaluation of biopsy H&E tissue sections performed by a blinded pathologist (S.J.D.)

	Low Dose (15 mg/kg)		High Dose (30 mg/kg)	
	-Cancer	+Cancer	-Cancer	+Cancer
-Red FL	22	7	20	7
+Red FL	4	13	5	15
PPV % (95%CI)	76.5% (50.1 – 93.2)		75.0% (50.9 – 91.3)	
NPV % (95%CI)	75.9% (56.5 – 89.7)		74.1% (53.7 – 88.9)	
Sensitivity % (95%CI)	65.0% (40.8 – 84.6)		68.2% (45.1 – 86.1)	
Specificity % (95%CI)	84.6% (65.1 – 95.6)		80.0% (59.3– 93.2)	
DOR (95%CI)	13.6 (3.0 – 62.0)		8.6 (2.3 – 32.4)	

*FL* fluorescence, *PPV* positive predictive value, *NPV* negative predictive value, *CI* confidence interval, *DOR* diagnostic odds ratio, *N/A* not applicable

**Fig. 3:**
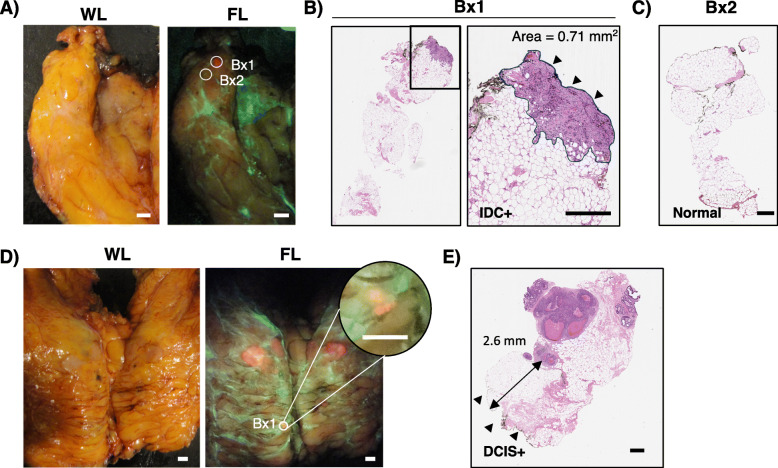
Detection of grossly occult sub-millimeter red fluorescence tumor foci. **A** WL and fluorescence images of a slice containing no clinically obvious disease from a patient who received 30 mg/kg 5-ALA HCl. Biopsies were collected in an area of focal red PpIX fluorescence (Bx1) and an adjacent area lacking PpIX fluorescence (Bx2). **B** H&E-stained longitudinal section of the Bx1 biopsy identified in **A**, which was determined to contain invasive ductal carcinoma by a blinded pathologist (S.J.D.). The imaged surface of the biopsy is indicated by the arrowheads. The area of tumor near the imaged surface measured 0.71 mm^2^. **C** H&E-stained longitudinal section of the Bx2 biopsy identified in **A**, which was determined to be negative for tumor by a blinded pathologist (S.J.D.). **D** White light and fluorescence images of slices of a lumpectomy from a patient who received 15 mg/kg 5-ALA HCl. A biopsy (Bx1) was collected from a small area of red fluorescence (inset digitally zoomed). **E** H&E-stained longitudinal section of the Bx1 identified in **D**, which was determined to contain DCIS > 2 mm below the imaged surface (arrowheads). Scale bar = 0.5 mm (A, D), 500 μm (B, C, E). WL, white light; FL, fluorescence; Bx, biopsy; IDC, invasive ductal carcinoma; DCIS, ductal carcinoma in situ

Accurate discrimination of small tumor foci was demonstrated by microscopic examination of H&E-stained biopsies collected from a small focus (~ 2 mm diameter) of red fluorescence and nearby non-red fluorescence tissue (Fig. 3A). Fluorescence imaging detected a 0.71-mm^2^ IDC focus (Fig. 3B) within surrounding normal breast tissue (Fig. 3C), otherwise occult to the pathologist (S.J.D.). In another specimen, DCIS 2.6 mm below the imaged tissue surface was visualized as a small area of red fluorescence (Fig. 3D, E). These data demonstrate the sub-millimeter fluorescence image resolution of our device, the cancer cell-selective nature of 5-ALA, and its ability to detect sub-surface disease.

**Fig. 4:**
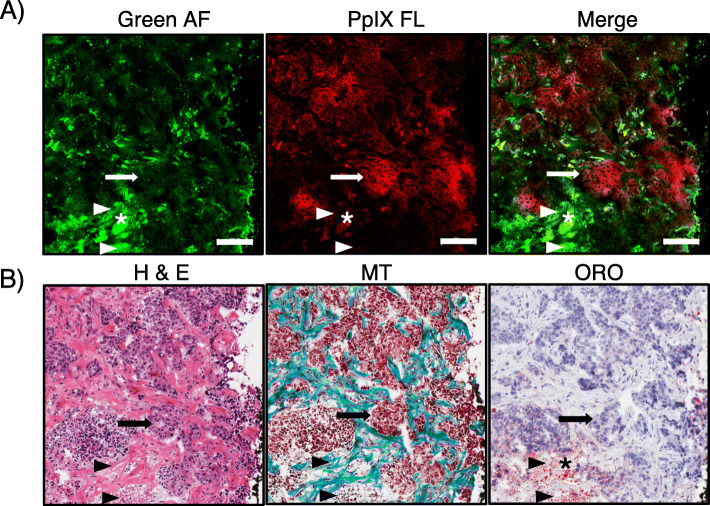
Cancer cell-specific localization of ALA-induced PpIX fluorescence. Representative **A** fluorescence microscopy and **B** corresponding histological images (bottom panel) from a biopsy collected inside the demarcated primary tumor boundary of a patient who received 30 mg/kg 5-ALA. The biopsy appeared red fluorescent with PRODIGI imaging. **A** Fluorescence microscopy was performed on cryosections cut from tumor core biopsies followed by **B** H&E, Masson’s Trichrome (MT), and Oil Red O (ORO) staining. Arrowheads depict green AF that was consistently observed in fibrous collagen tissue and in locations of necrosis, as confirmed with H&E and MT staining. Adipose tissue, identified by ORO staining, demonstrated both green and red AF (asterisk). PpIX fluorescence microscopic imaging confirmed cancer cell localization of PpIX (arrow), which was not observed during green AF imaging. Scale bar = 100 μm

Confocal fluorescence microscopy of frozen tumor tissue sections from representative patients was used to confirm the cancer-specific localization of PpIX and characterize healthy tissue AF. Fluorescence microscopy showed bright PpIX fluorescence in the cytoplasm of cancer cells identified in corresponding H&E-stained tissue sections (Fig. 4A). No significant red PpIX fluorescence was observed in the cancer stroma, which exhibited bright green AF (Fig. 4A) due to dense connective tissues, such as collagen and elastin, visible in Masson’s trichrome-stained section (Fig. 4B) [68]. Adipose tissue, which was identified using Oil Red O-stained sections, was visible in both the green and red channels, demonstrating broad green-to-red AF albeit with a qualitatively subdued intensity compared with the green AF of connective tissues or the red PpIX fluorescence. The boundary between cancer foci containing PpIX fluorescence and connective tissue green AF was distinct at the cellular level (Fig. 4), indicating the specificity of PpIX accumulation in cancer cells compared with healthy surrounding tissues.

### Characterization of normal and cancerous breast tissue fluorescence

**Fig. 5:**
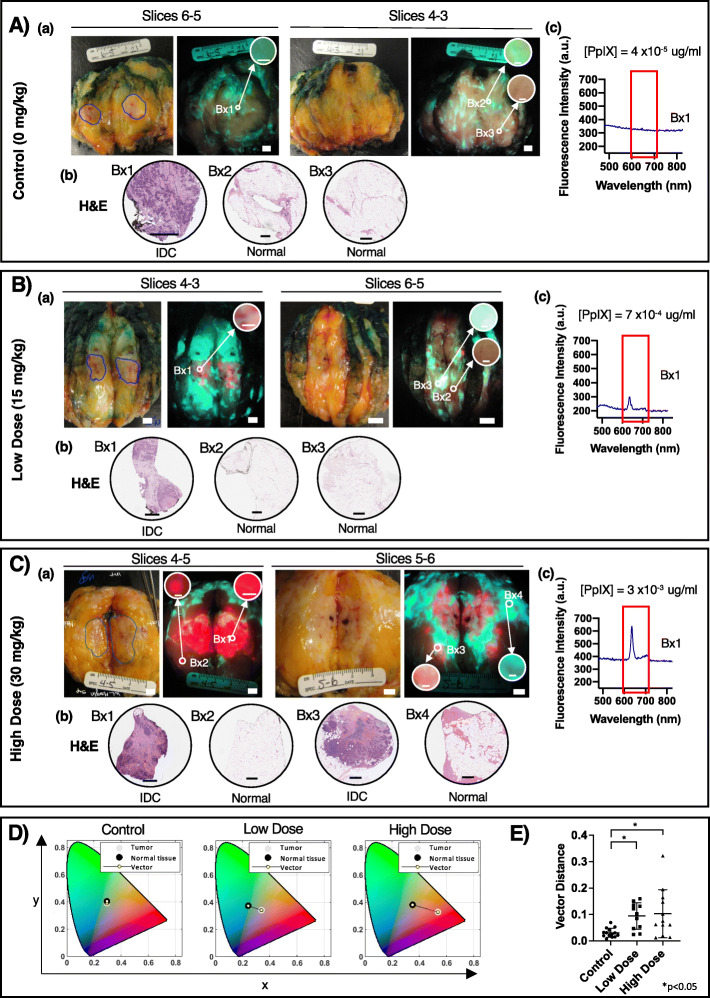
Ex vivo breast specimen fluorescence in patients with and without 5-ALA. Representative white light and fluorescence images with corresponding biopsy-based H&E and fluorescence spectra from patients with invasive ductal carcinoma receiving **A** no 5-ALA, **B** 15 mg/kg 5-ALA, or **C** 30 mg/kg 5-ALA. (a) Biopsies were collected in areas inside (Bx1) the PA demarcated tumor (blue line) and outside the demarcated tumor (Bx2-4) on an adjacent slice of the specimen. Circular insets are digitally magnified images of the biopsy areas demonstrating the fluorescence color. (b) H&E-stained longitudinal biopsy sections were examined by a blinded pathologist (S.J.D.) for the presence of cancer. (c) Point spectroscopy was performed at the Bx1 location and smoothed fluorescence spectra in the region of PpIX emission (red box, 635 nm peak) are presented. **D** Representative chromaticity diagrams (CIE xyY displaying the average pixel color inside the demarcated tumor border and outside to normal tissue contrast from fluorescence images of specimens from patients described in parts **A**–**C** of this figure. **E** Bar graph depicting the average vector distance between the average pixel color of the primary tumor and surrounding normal tissue. * p < 0.05, one-way ANOVA with multiple comparisons. Scale bar = 5 mm (a, white light and fluorescence images), 100 μm (a, inset), 500 μm (b, H&E sections). Bx, biopsy; IDC, invasive ductal carcinoma

To characterize fluorescence features of cancer and normal breast tissues, focal areas inside and outside the demarcated tumor border were visually appraised before biopsy and categorized by a research team member as red, green, or dull pink/brown fluorescence during biopsy (Fig. 5A(a), B(a), C(a)). H&E-stained sections were evaluated for carcinoma and other normal breast tissues (Fig. 5A(b), B(b),C(b)). In patients receiving 5-ALA, biopsies from outside the tumor border were obtained from areas of red PpIX fluorescence, if present, while the remaining biopsies were from areas identified as green or pale pink/brown fluorescence. In control and both 5-ALA dose groups, areas of bright green fluorescence on gross imaging corresponded to connective tissues (Fig. 5A–C), while pale pink/brown areas were predominately adipose tissue (Fig. 5A, B). Quantitative fluorescence point spectroscopy performed at biopsy locations within the demarcated tumor border confirmed PpIX (peak emission 635 nm) in red fluorescence tumors of patients that received 5-ALA (Fig. 5B(c) and C(c)) but not in controls (Fig. 5A(c)). Among both 5-ALA groups, histological assessment of H&E tissue sections of biopsies from red fluorescence tumors showed that while fluorescence imaging of the gross specimen often appeared homogeneously bright red in the demarcated primary tumor [due to increased PpIX in cancer cells (Fig. 5C(a), Bx1)], in fact, microscopically, these corresponding tissue regions contained a mixture of cancer and healthy tissue cells (Fig. 5C(b), Bx1). Taken together with our fluorescence microscopy data (Fig. 4), which demonstrated specific accumulation of PpIX in cancer cells and not in interspersed normal tissue, these data demonstrate that visualization based on PpIX red fluorescence is feasible in areas with a mixture of tissue types (malignant, connective and adipose). Additionally, fluorescence imaging revealed the multifocal and spatially heterogeneous nature of invasive breast cancers (Fig. 5C(a), slices 5–6).

To determine the effect of different 5-ALA doses on cancer fluorescence contrast compared with normal tissues, fluorescence images were analyzed in the CIE xyY (chromaticity-luminance) color space, which is more sensitive to differences in fluorescence images than RGB intensity-based analysis [69]. For a given image, the average pixel color (x,y coordinates) in two regions of interest (ROI) was calculated and the Eucliden distance between these points was measured (Fig. 5D). The average (±SD) Euclidean distance for the low-dose (0.09 ± 0.04) and high-dose (0.10 ± 0.09) 5-ALA groups was significantly greater than the control (0.03 ± 0.2, p < 0.05, one-way ANOVA with Tukey’s multiple comparison test), confirming the lack of visually perceptible cancer fluorescence contrast in the control group and the improved fluorescence contrast of PpIX red fluorescence cancers relative to healthy background tissue in the 5-ALA groups.

### Effect of tissue composition on detection of PpIX fluorescence

The average percentage of the total biopsy area classified as cancer or connective tissue in histologically confirmed cancer cell-positive biopsies was compared between biopsies collected from red fluorescence (true positive) or green fluorescence (false negative) regions (Supplementary Fig. 5). Given that the 5-ALA dose did not significantly affect the classification of biopsies (comparable number of TP and FN were observed between both 5-ALA dose groups), the data from both dose groups was pooled. While the average percent cancer was higher in biopsies collected from red versus green fluorescence regions, this was not statistically significant (Supplementary Fig. 5A, 24.43% vs 14.82%, p = 0.12). Conversely, the average percent connective tissue was significantly lower in biopsies collected from PpIX red versus green fluorescence regions (Supplementary Fig. 5B, 56.04% vs 73.18%, p = 0.007). PRODIGI simultaneously detects green and red fluorescence to produce a composite color image; therefore, the relative amount of both connective and cancer tissue is hypothesized to affect the resultant fluorescence color in that region of tissue. The carcinoma-to-connective tissue ratio (area of a biopsy tissue section classified as cancer divided by the area classified as connective tissue) demonstrates that histologically confirmed cancer cell-positive biopsies collected from red fluorescence regions had a significantly higher carcinoma-to-connective tissue ratio compared to those classified as green fluorescence (Supplementary Fig. 5C, 0.49 vs 0.20, p = 0.02).

### Safety and feasibility of intraoperative imaging

No adverse drug reactions or device-related adverse events were observed during the trial. One patient, who reported a sunburn after post-operative discharge, did not adhere to the sun protection instructions. Intraoperative imaging of the surgical cavity was performed in 30 study participants (n = 10/group) to demonstrate technical feasibility, safety, and integration of PRODIGI into the BCS clinical workflow (Supplementary Fig. 6). We noted that the fixed placement of LEDs did not adequately illuminate the entire cavity surface homogeneously. To overcome this, we scanned the surface in a sweeping manner with the device and collected videos of the fluorescence. Cavity imaging integrated into the standard operating room workflow without significant disruption. Approximately 1–2 min was required to perform a complete scan with real-time video display of all anatomical margins of the surgical cavity. The device was quickly and easily draped prior to entering the sterile field and was thermally stable during operation and comfortable for the operator. Furthermore, the device was used to image surgical specimens without workflow disruption in the pathology setting.

## Discussion

We present preliminary evidence of effectiveness from a Phase II RCT demonstrating the first clinical use of 5-ALA plus PRODIGI, a prototype fluorescence imaging device, for real-time visualization of breast cancers. The primary objective of this study was to determine estimates of diagnostic accuracy including PPV, NPV, sensitivity, specificity, and DOR for the visualization of breast cancer using 5-ALA-induced PpIX fluorescence plus PRODIGI. We also evaluated the technical feasibility, safety, and clinical workflow integration of this approach in the intraoperative setting. To the best of our knowledge, this is the first RCT to test a *fully* handheld, wide-field fluorescence imaging device for real-time intraoperative breast cancer imaging of lumpectomy and mastectomy specimens and surgical cavities, as well as the first to report results for 15 and 30 mg/kg 5-ALA-induced PpIX fluorescence in breast cancer patients.

Surgical margins are a significant challenge in the treatment of solid cancers [11]. During this study the Society of Surgical Oncology/American Society Radiation Oncology/American Society of Clinical Oncology released joint guidelines for BCS recommending a standard definition of a positive margin based on clinical evidence [6, 70]. The guidelines define positive margins as “ink on tumor” for invasive breast cancer [6] and cancer within 2 mm of the inked margin for DCIS [70]. Surgeons disagree regarding the recommendations [19, 71, 72] and the effect of the guidelines on re-excision rates varies [71, 73–79]. Many studies still report suboptimal re-excision rates [71, 74, 76, 79–81], above the internationally accepted target of 10% [7, 22], reinforcing the need for new imaging methods that allow surgeons and pathologists to visualize grossly occult disease on the lumpectomy and in the surgical cavity during index surgery.

5-ALA was well tolerated and enabled visualization of grossly obvious tumors and occult disease across all tumor grades (I-III) and types, including IDC and ILC with and without in situ disease. Both doses tested were lower than the only dose previously reported for clinical use in breast cancer (40 mg/kg) [48] and consistently induced bright red cancer fluorescence (contrasted against normal tissue AF) detected by the device.

PRODIGI was easy to use and practical due to its handheld form factor (e.g., non-cart based), which overcomes the large footprint limitation of existing closed- and wide-field fluorescence surgical imaging systems. The device was safely integrated into the standard surgical workflow in both the operating room and the pathology suite without affecting standard specimen processing procedures. PpIX fluorescence imaging with PRODIGI was feasible in the presence of blue dye, which is commonly used in sentinel node mapping. Although there is a potential for the blue dye to absorb light in the red wavelength range, PpIX was visualized in tissues stained with blue dye. PRODIGI imaging within a small lumpectomy cavity was impeded by the form factor of the prototype which led to shadow artefacts due to the position of the LEDs. Additionally, although a standardized imaging distance of ~ 10 cm was maintained when imaging excised tissue on the bench, achieving consistent distance for intracavity imaging was challenging. The next-generation device, currently being developed by our group, is anticipated to overcome these challenges by improving the optical power density output, re-configuring the optical components to maximize cavity and specimen surface illumination, including range finder technology to ensure a standardized working distance is maintained for optimal imaging and re-designing the form factor. For the free-hand imaging modality of the PRODIGI device, there is a potential for images taken to be blurry. We acknowledge that some of our images (Fig. 2A(b) and Fig. 2B(d)) are blurry and were not frames from a video. A blurry image, however, did not detract from real-time visualization of PpIX fluorescence. The next-generation device will include a tap-to-focus feature. A multicenter Phase III interventional imaging RCT will use this optimized device to evaluate the effectiveness of 5-ALA-induced PpIX fluorescence image-guided margin assessment and resection during BCS. Future studies may also evaluate fluorescence imaging during gross examination of specimens to facilitate rapid, targeted specimen sectioning and identification of areas for touch-prep cytology and frozen-section analysis, to improve rapid decisions about additional tissue excision and location of positive margins in the surgical cavity.

Predictive values depend on disease prevalence, with a higher disease prevalence associated with higher PPV and lower NPV, and vice versa [82]. Analysis was, therefore, stratified by biopsy location as the prevalence of cancer cells is higher inside versus outside the tumor border. In our study, high PPV inside and high NPV outside the tumor border were observed for both 5-ALA doses, indicating that PRODIGI functioned as expected. However, sensitivity and specificity are generally accepted to not be influenced by differences in disease prevalence [83]. Thus, while these calculations are possible with biopsies from inside and outside the tumor border, more meaningful sensitivity and specificity are derived without stratification by biopsy location. When compared to MarginProbe, which uses non-imaging radiofrequency signals to alert surgeons about positive margins [84–86], our platform may be less likely to result in removal of cancer-negative tissues. Moreover, our approach is more sensitive and comparably specific to conventional BCS [86].

False positives and negatives were seen in both 5-ALA dose groups inside and outside the demarcated tumor border. False positives were only observed in 5-ALA patients (outside the tumor border), and not in controls, indicating likely ALA-induced PpIX production in non-malignant cells. The majority of false positive biopsies (5/9), examined histologically, demonstrated non-malignant proliferative changes (hyperplasia, columnar cell change, atypical ductal hyperplasia, sclerosing adenosis, and fibrocystic change) with infiltrating immune cells present in one biopsy. If we consider the detection of these “abnormal” proliferative changes as a desirable outcome of the test, the PPV to detect abnormal breast tissue (including malignant and non-malignant proliferative changes) increases to 89% and 70% for the low- and high-dose groups, respectively. Other studies using 5-ALA have observed the accumulation of PpIX fluorescence in premalignant diseases [57], in areas with immune cell infiltration [87, 88] and in benign disease, such as Grade 1 meningiomas [89]. Removing non-malignant tissues that are associated with increased breast cancer risk [90] may outweigh the potential effect on cosmesis during BSC, minimizing the clinical impact of false positives. Unlike other non-targeted approaches, such as circumferential cavity shaving [81], fluorescence imaging would conceivably allow for more spatially targeted revision of margins, reducing unnecessary removal of additional tissue which is desirable in optimizing cosmesis. Nevertheless, additional studies are needed to understand how 5-ALA behaves in benign and malignant breast tissues to improve their differentiation during intraoperative fluorescence imaging.

Eleven of the 14 false negative biopsies were collected from inside the tumor border, the majority of which (8/11) came from primary tumors that had observable red fluorescence elsewhere inside the demarcated tumor or in an area contiguous with the demarcated tumor on an adjacent slice, suggesting heterogeneity in biological factors that may influence 5-ALA uptake and the production, accumulation, and/or detection of PpIX in the primary tumor. For example, differences in tissue composition can affect optical properties and light transport in tissue which can affect the detection of PpIX. Moreover, endogenous fluorophores that emit high-intensity green AF (collagen, flavin adenine dinucleotide-FAD, reduced nicotinamide adenine dinucleotide-NADH) [91] may “mask” PpIX fluorescence, especially in small cancer foci.

Multifocal disease, associated with poorer disease-free survival [92], was also visualized in serially sectioned specimens. PpIX fluorescence revealed lobulated tumor borders and heterogenous tumor composition, which was consistent with patients’ preoperative imaging/pathology but not appreciable by palpation. While some specimens had uniform bright red fluorescence tumors, others had heterogeneous (multifocal) bright red fluorescence foci with green connective tissue interspersed throughout the tumor, which is consistent with carcinoma-associated fibrosis [93]. We observed spatial heterogeneity in the density of fluorescent connective tissues in the surgical specimens during macroscopic examination with some specimens being homogeneously bright green and others showing a more mottled appearance. Moreover, increased microvasculature may preferentially absorb the 405 nm excitation light and emitted PpIX fluorescence. These findings may aid decision-making for oncoplastic surgical techniques [94] and identify patients with residual or multicentric disease beyond the primary tumor.

Limitations of the study include its single-center design, variability in the time to imaging, restriction on the total number of biopsies collected per patient, and a focus on visualization of carcinoma inside the serially sectioned specimen and not on the specimen margins. PMCC draws patients from various regional community practices representative of patients seen throughout Canada and the U.S. However, clinical practice by breast surgeons (A.M.E., W.L.L.) in our academic setting may vary from community practices [95]. Variability in the imaging time relative to administration of 5-ALA between patients is unavoidable in a surgical intervention study at a high-volume center. Nevertheless, we aimed to administer 5-ALA 2-4 h before anesthesia, to enable imaging between 2.5 and 5 h post-administration, which is clinically relevant for maximal PpIX signal [96]. The institution restricted the number and size of biopsies collected from inside and outside the demarcated tumor border. To maximize the number of PpIX red fluorescence areas analyzed histologically, biopsies were collected under fluorescence image guidance, possibly leading to a sampling selection bias. However, given the restrictions on biopsy collection, it would be difficult to collect enough biopsies without fluorescence image guidance to calculate sensitivity outside the tumor border. Lastly, future studies will assess the accuracy of 5-ALA-induced fluorescence visualization for the detection of positive margins during BCS.

## Conclusions

Intraoperative fluorescence imaging is an emerging technique with significant potential to improve clinical outcomes in breast cancer patients. The superficial (~ 2 mm [56]) penetration of PRODIGI’s 405 nm excitation light is more suitable for intraoperative margin assessment compared to NIR fluorescence imaging methods, which can image to depths of several centimeters [27, 28, 97, 98] and are, therefore, more likely to result in false positive margin readings. Unlike other non-image-based methods, including the FDA-approved MarginProbe [86], our approach produces a real-time color image of the anatomical location and extent of carcinoma with sub-millimeter resolution. The differing emission spectra of normal tissue AF and PpIX are visually distinguishable, providing excellent fluorescence contrast of the cancers against background healthy tissues and simplifying fluorescence image interpretation for the operator [99]. This facilitates real-time decision-making by guiding surgeons to potential areas of microscopic disease within a macroscopic setting [99], and enabling treatment plan adjustments, which is not possible with current preoperative imaging modalities.

Our study also contributes to the growing clinical evidence of 5-ALA for intraoperative visualization of clinically occult cancer beyond neurosurgery where this approach is becoming the standard of care [43]. The versatile design of the novel handheld fluorescence imaging device could help to translate this approach to other cancer surgeries where margin assessment and image-guided resection are valuable. Future clinical adoption will be predicated on safe and effective device and contrast agent combinations that are easily integrated into the operating room workflow, meet the user’s needs (wireless one-handed use), and are cost-sensitive.

## Supplementary Information

**Additional file 1: Supplementary Figure 1.** Study Workflow Diagram.

**Additional file 2: Supplementary Figure 2.** Prototype Handheld Fluorescence Imaging Device and Benchtop Phantom.

**Additional file 3: Supplementary Figure 3.** Red fluorescence detected in tumor of patient without 5-ALA.

**Additional file 4: Supplementary Figure 4.** MacAdam’s color discrimination ellipses.

**Additional file 5: Supplementary Figure 5.** Effect of tissue composition on PpIX detection.

**Additional file 6: Supplementary Figure 6.** Draping and imaging with the PRODIGI device in the operating room.

**Additional file 7: Supplementary Table 1.** Statistical comparison of diagnostic accuracy measures for low and high dose 5-ALA.

## Data Availability

Data generated in this manuscript are available on request from the corresponding author (RSD) to comply with the Princess Margaret Cancer Center Institute ethics regulations to protect patient privacy. All requests will be promptly reviewed by the UHN Technology Development and Commercialization team to verify whether the request is subject to any intellectual property or confidentiality obligations. Any data and materials that can be shared will be released subject to a data transfer agreement.

## Abbreviations

5-ALA HCl: 5-Aminolevulinic acid hydrochloride
AF: Autofluorescence
BCS: Breast-conserving surgery
CT: Computed tomography
DCIS: Ductal carcinoma in situ
DOR: Diagnostic odds ratio
FIGS: Fluorescence image-guided surgery
H&E: Hematoxylin and eosin
IDC: Invasive ductal carcinoma
ILC: Invasive lobular carcinoma
LED: Light-emitting diode
LR: Local recurrence
MT: Masson’s trichrome
NIR: Near infrared
NPV: Negative predictive value
ORO: Oil Red O
PA: Pathologist’s assistant
PET: Positron emission tomography
PpIX: Protoporphyrin IX
PPV: Positive predictive value
qF: Quantitative fluorescence
RCT: Randomized controlled trial
WL: White light
