# Supplementary figures and images for: Intraoperative fluorescence imaging with aminolevulinic acid detects grossly occult breast cancer: a phase II randomized controlled trial
Source: Breast Cancer Res. 2021 Jul 12;23:72. doi: 10.1186/s13058-021-01442-7 (PMC8276412; doi:10.1186/s13058-021-01442-7)

# Supplementary Figure 1

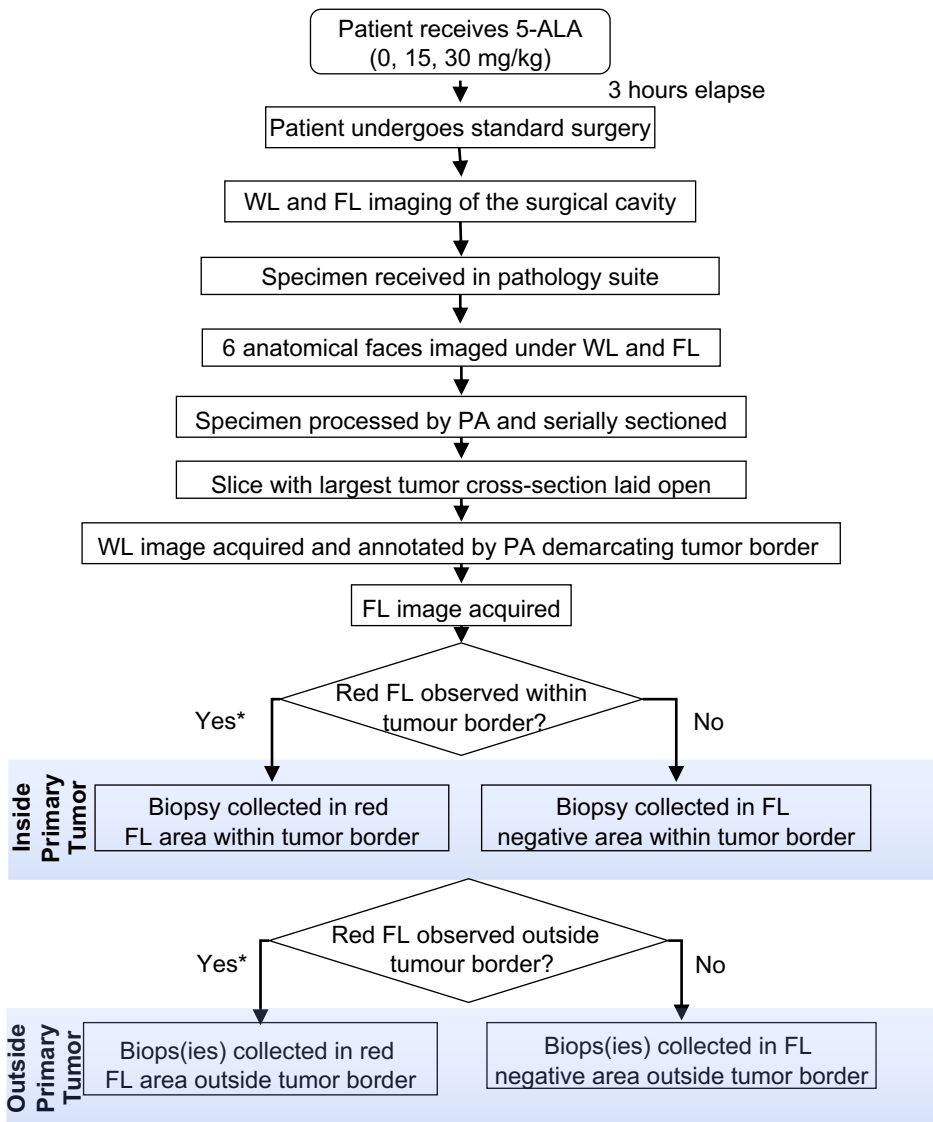

Supplement: Supplementary file 1 — Additional file 1: Supplementary Figure 1. Study Workflow Diagram. [file 13058_2021_1442_MOESM1_ESM.pdf]

# Supplementary Figure 2

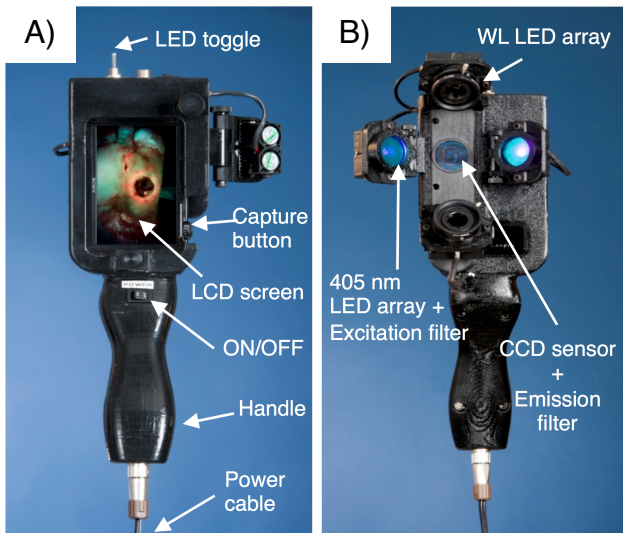

Supplement: Supplementary file 2 — Additional file 2: Supplementary Figure 2. Prototype Handheld Fluorescence Imaging Device and Benchtop Phantom. [file 13058_2021_1442_MOESM2_ESM.pdf]

Supplementary Figure 3

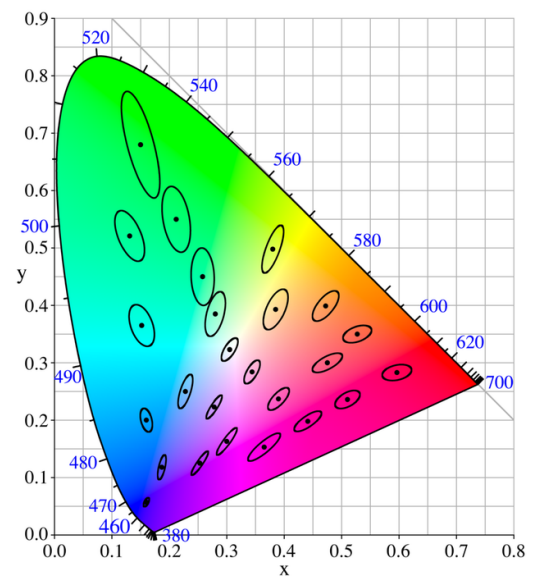

Supplement: Supplementary file 3 — Additional file 3: Supplementary Figure 3. Red fluorescence detected in tumor of patient without 5-ALA. [file 13058_2021_1442_MOESM3_ESM.pdf]

Supplementary Figure 4

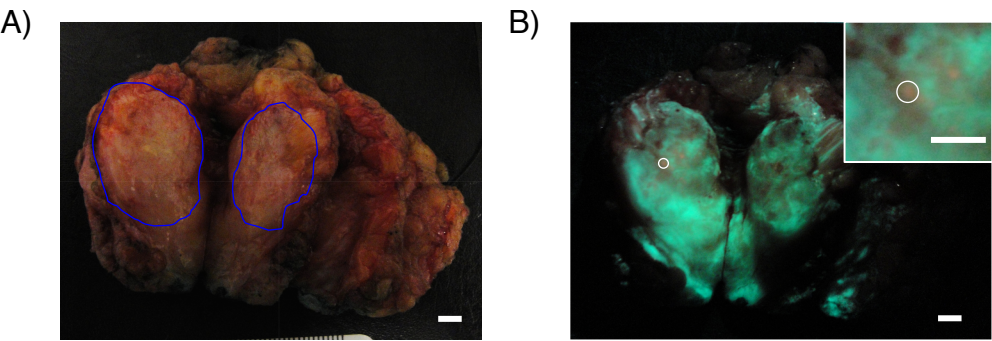

Supplement: Supplementary file 4 — Additional file 4: Supplementary Figure 4. MacAdam’s color discrimination ellipses. [file 13058_2021_1442_MOESM4_ESM.pdf]

# Supplementary Figure 5

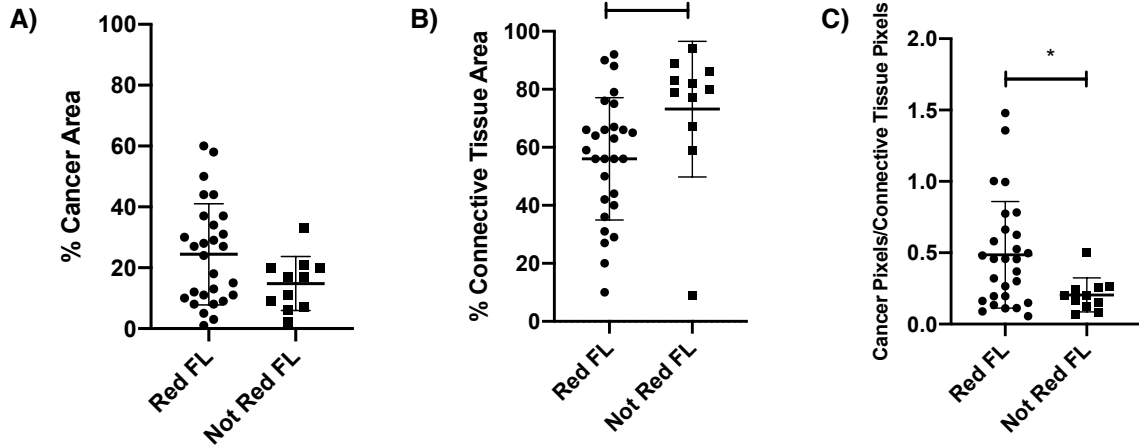

Supplement: Supplementary file 5 — Additional file 5: Supplementary Figure 5. Effect of tissue composition on PpIX detection. [file 13058_2021_1442_MOESM5_ESM.pdf]

# Supplementary Figure 6

A)

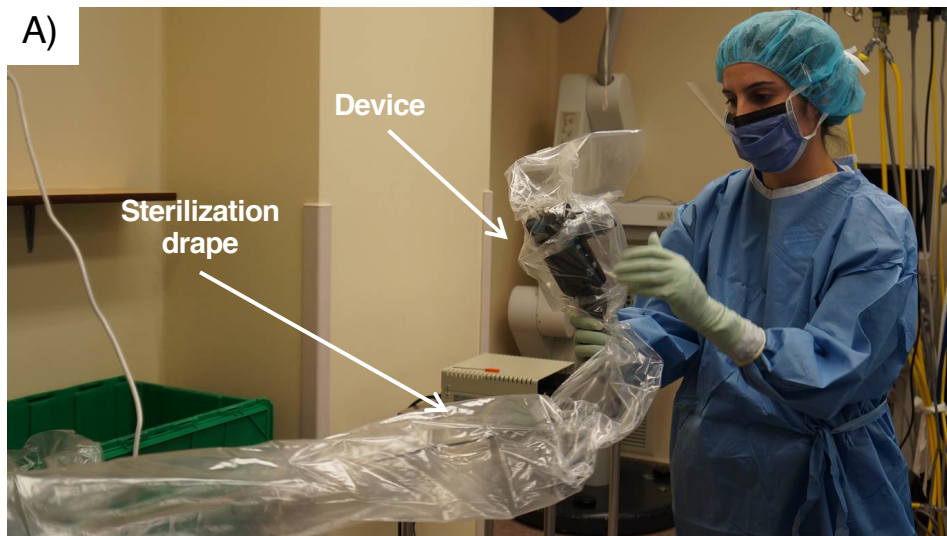

B)

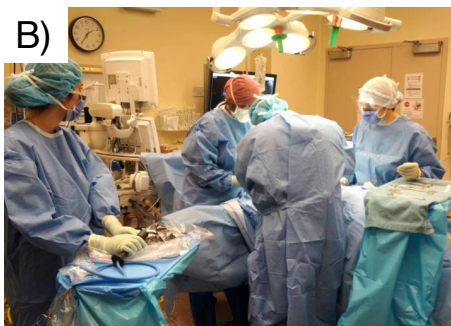

C)

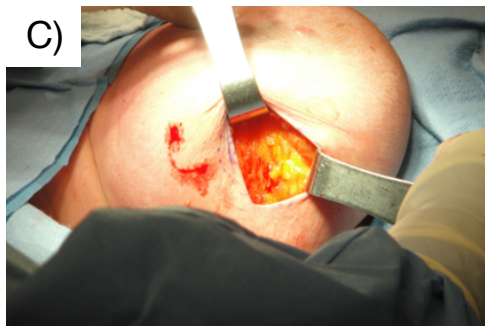

D)

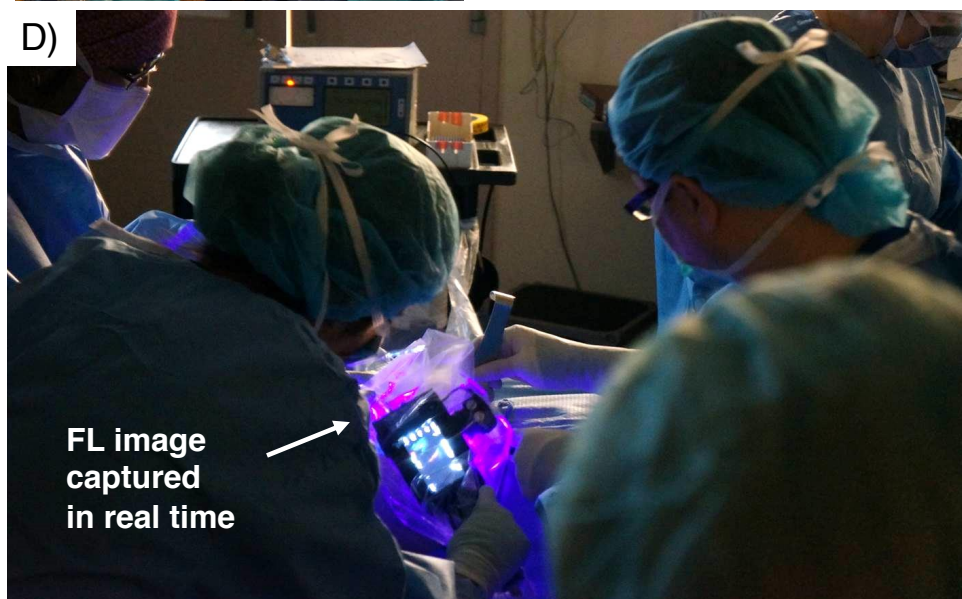

Supplement: Supplementary file 6 — Additional file 6: Supplementary Figure 6. Draping and imaging with the PRODIGI device in the operating room. [file 13058_2021_1442_MOESM6_ESM.pdf]
