# Supplementary material for: Intraoperative fluorescence imaging with aminolevulinic acid detects grossly occult breast cancer: a phase II randomized controlled trial
Source: Breast Cancer Res. 2021 Jul 12;23:72. doi: 10.1186/s13058-021-01442-7 (PMC8276412; doi:10.1186/s13058-021-01442-7)
Supplement: Supplementary file 7 — Additional file 7: Supplementary Table 1. Statistical comparison of diagnostic accuracy measures for low and high dose 5-ALA. [file 13058_2021_1442_MOESM7_ESM.docx]

**SUPPLEMENTARY TABLE 1**

|  | p-value  Low Dose (15 mg/kg) vs. High Dose (30 mg/kg) | | |
| --- | --- | --- | --- |
|  | Inside the tumor border | Outside the tumor border | Locations Combined |
| PPV | 1 | 1 | 1 |
| NPV | 1 | 1 | 1 |
| Sensitivity | 0.71 | 1 | 1 |
| Specificity | 1 | 1 | 0.73 |
| DOR | N/A | 0.54 | 0.86 |
